# Sponges and Their Symbionts as a Source of Valuable Compounds in Cosmeceutical Field

**DOI:** 10.3390/md19080444

**Published:** 2021-08-02

**Authors:** Roberta Esposito, Nadia Ruocco, Thomas Viel, Serena Federico, Valerio Zupo, Maria Costantini

**Affiliations:** 1 Department of Marine Biotechnology, Stazione Zoologica Anton Dohrn, Villa Comunale, 80121 Naples, Italy; roberta.esposito@szn.it (R.E.); nadia.ruocco@szn.it (N.R.); serena.federico@studenti.unina.it (S.F.); 2 Department of Biology, University of Naples Federico II, Complesso Universitario di Monte Sant’Angelo, Via Cinthia 21, 80126 Naples, Italy; 3 Stazione Zoologica Anton Dohrn, Department of Marine Biotechnology, Villa Dohrn, Punta San Pietro, 80077 Naples, Italy; thomas.viel7@gmail.com

**Keywords:** sponges, bacteria, fungi, anti-oxidant, anti-aging, skin whitening, anti-microbial, photoprotection

## Abstract

In the last decades, the marine environment was discovered as a huge reservoir of novel bioactive compounds, useful for medicinal treatments improving human health and well-being. Among several marine organisms exhibiting biotechnological potential, sponges were highlighted as one of the most interesting phyla according to a wide literature describing new molecules every year. Not surprisingly, the first marine drugs approved for medical purposes were isolated from a marine sponge and are now used as anti-cancer and anti-viral agents. In most cases, experimental evidence reported that very often associated and/or symbiotic communities produced these bioactive compounds for a mutual benefit. Nowadays, beauty treatments are formulated taking advantage of the beneficial properties exerted by marine novel compounds. In fact, several biological activities suitable for cosmetic treatments were recorded, such as anti-oxidant, anti-aging, skin whitening, and emulsifying activities, among others. Here, we collected and discussed several scientific contributions reporting the cosmeceutical potential of marine sponge symbionts, which were exclusively represented by fungi and bacteria. Bioactive compounds specifically indicated as products of the sponge metabolism were also included. However, the origin of sponge metabolites is dubious, and the role of the associated biota cannot be excluded, considering that the isolation of symbionts represents a hard challenge due to their uncultivable features.

## 1. Introduction

Marine sponges represent a fascinating phylum of marine invertebrates, hosting a wide symbiotic community together with a huge production of secondary metabolites [1,2,3,4,5,6,7]. The sponge-associated biota may bring together a broad group of phylogenetic lineages, including archaea, bacteria, and fungi [8,9]. The relationships between sponges and their mutualistic symbionts are complex, and the production of bioactive secondary metabolites might have a possible defense role or be involved in the competition for space within benthic habitats [10,11]. On the whole, sponge symbionts were recognized to be responsible for the host metabolism and growth, chemical defense, and adaptation to biotic and abiotic stressors [2,12,13,14].

The discovery of marine bioactive metabolites as potential drugs for the pharmaceutical, nutraceutical, and cosmeceutical industries prompted several research projects relying on the identification of novel chemical moieties with innovative biological functions [15]. Recently, the cosmeceutical field has been fast-growing, since consumers have given greater attention to creams and lotions containing natural compounds with pharmacological properties [16]. Cosmeceuticals are topical products containing some bioactive ingredients that mimic drug-like benefits by enhancing skin health-related function [16,17]. On a global scale, the cosmeceutical industry is gradually shifting to natural compounds for their biocompatible, safe, and eco-friendly properties [18]. The success of cosmeceutical productions primarily depends on safety; low costs; and the ability to maintain the active ingredient, deliver it in a biologically active form, and exert a biological effect through known mechanisms [19]. To overcome these latter issues, particularly related to unsuitable chemical properties, some encapsulation and nano-formulation methods were developed to greatly improve drug delivery and effectiveness [20,21,22,23,24,25].

**Figure 1:**
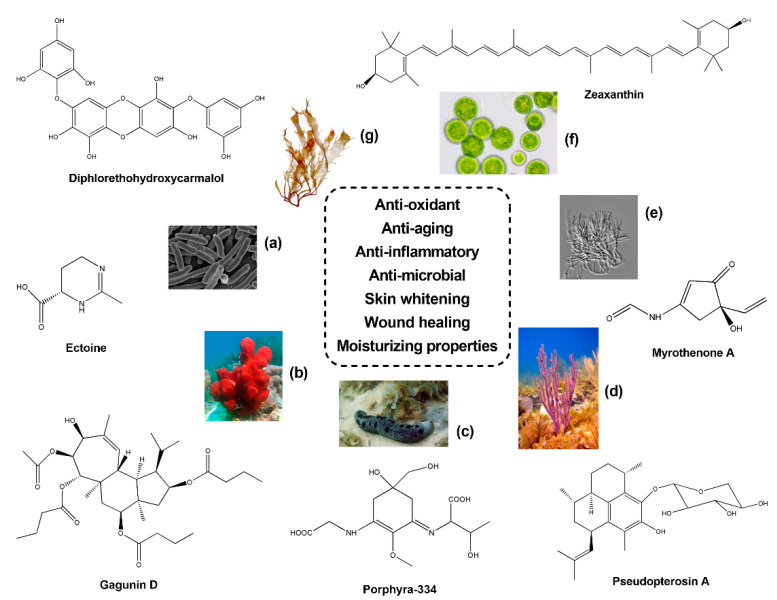
Examples of bioactive compounds isolated from bacteria (**a**), sponges (**b**), echinoderms (**c**), corals (**d**), fungi (**e**), micro- (**f**) and macro-algae (**g**) reported as suitable candidates for the formulation of cosmetics.

Despite cosmeceuticals being historically retrieved from terrestrial plants [26,27,28], in the last decades, several of them were discovered in marine environments. In fact, the ocean represents a rich source of bioactive ingredients, such as vitamins, minerals, amino acids, proteins, lipids, polysaccharides, terpenoids, polyphenols, pigments, and enzymes, which find several applications in the cosmeceutical field [29]. Marine cosmeceuticals showed a broad range of beneficial activities, such as anti-oxidants, anti-UV, anti-aging, anti-tyrosinase (skin whitening), anti-microbial, wound healing, and emulsifying properties (Figure 1) [29,30,31,32,33,34,35,36,37,38,39,40,41,42,43]. 

Recently, much attention has been paid to marine anti-oxidants, including mycosporines and mycosporine-like amino acids (MAAs), carotenoids and other compounds exhibiting multiple roles within cosmeceutical field [44,45]. Some examples are pigments (e.g., carotenoids), extremely abundant in the marine environment since they are produced by all autotrophic organisms (e.g., bacteria, archaea, algae and fungi). Carotenoids include carotenes (e.g., lycopene and α- and β-carotene) and xanthophylls (e.g., astaxanthin, fucoxanthin and lutein), which showed anti-oxidant activities [46] protecting skin from Reactive Oxygen Species (ROS) that are normally released within the cells after the natural oxidation induced by UV radiation and skin aging [42]. Since synthetic compounds may exert toxic effects for human health and wellness [47], natural anti-oxidants were investigated for their potential use in cosmetics [48,49]. Anti-microbial and anti-fouling agents that protect against skin disease-related pathogens, such as *Staphylococcus epidermis*, *Staphylococcus aureus*, *Pseudomonas aeruginosa* and *Candida albicans*, were also described from various sources and considered useful tools for the formulation of cosmetic products and dermatological treatments [39,50,51,52,53,54]. Moreover, bioactive compounds with anti-tyrosinase activity found several applications in the cosmetic industry, since tyrosinase represents a key enzyme involved in melanin biosynthesis, and the block of its enzymatic activity might be used for skin whitening treatments, whose deployment is extremely popular in some countries [55]. Surfactants and emulsifiers, with both hydrophilic and hydrophobic groups, could also be used in the cosmetic field [56,57]. Several protein-polysaccharide complexes, glycolipids and lipopeptides isolated from marine microorganisms were studied for the production of biosurfactants and bioemulsifiers [58]. For instance, chitosan, due to its high water-binding capacity, was proposed as a skin moisturizer and delivery agent in cosmeceutical preparations of anti-aging products [59]. 

Recognized producers of marine cosmeceuticals are cyanobacteria, along with micro- and macro-algae [24,60,61,62,63], with several compounds under clinical trials or already approved for the market [64,65]. As mentioned before, sponge-associated microbiota produce a plethora of bioactive compounds with beneficial properties for human health [6]. Despite the great biotechnological relevance, so far, only a few studies have reviewed the potential applications of sponge symbiont metabolites in the cosmetic field focusing on specific sponge species [66] or grouping several taxa of marine organisms [29].

In the present review, we analyzed a collection of scientific literature on sponge symbiont-related compounds displaying interesting biological activities in the cosmeceutical field. In particular, we focused on bacteria and fungi, which are extremely abundant within sponge associated communities. Moreover, we also considered sponge metabolites, whose biological activities were found extremely suitable for cosmeceutical formulations.

## 2. Sponge Symbionts in Cosmeceutical Field

### 2.1. Bacteria

A variety of bioactive compounds described from marine bacteria such as polyketides, alkaloids, peptides, proteins, lipids, mycosporines and MAAs, glycosides, isoprenoids and hybrids, displayed surprising activities, such as photo-protective, anti-aging, anti-microbial, anti-oxidant, and moisturizing activities [58,67]. The interesting capability to produce some UV-absorbing compounds, including scytonemins (exclusively cyanobacteria), mycosporines, carotenoids and melanin, was explained through possible evolutionary mechanisms evolved to protect sponges from the harmful effects of UV radiation [68,69].

As reported in the introduction section, carotenoids, such as β-carotene and lycopene, exhibited a photoprotective activity, thus revealing several applications in cosmeceutical fields [70]. Dharmaraj and co-authors [71] investigated the carotenoid extract of a *Streptomyces strain* (AQBWWS1) associated to the sponge *Callyspongia diffusa* collected from the west coast of Kerala (India). Its chemical profile revealed the presence of lycopene, suggested as a potential ingredient for the preparation of cosmetic products [71].

A novel diapolycopenedioic acid xylosyl ester A, extracted from the marine sponge- derived bacterium *Rubritalea squalenifaciens* sp. nov., revealed a potent anti-oxidant activity in a ^1^O_2_ suppression model with half maximal inhibitory concentration (IC_50_) of 4.1 µg/mL [72]. The alkaloid Diazepinomicin was also isolated from the strain *Micromonospora* sp. RV115 associated to the sponge *Aplysina aerophoba* collected from the Mediterranean Sea. This molecule was able to protect the human kidney (HK-2) and human promyelocytic (HL-60) cell lines from toxicity and genomic damage induced by H_2_O_2_ [73]. The metabolites isolated from *Virgibacillus* sp. associated to the sponge *C. diffusa* (Gulf of Mannar) showed 1,1-Diphenyl-2-Picrylhydrazyl (DPPH) radical scavenging activity with IC_50_ of 857.49 µg/mL. In addition, a clear hydroxyl and superoxide free radical scavenging activity was detected (IC_50_ = 471.07 µg/mL and 1353.28 µg/mL, respectively), probably correlated to the presence of bioactive compounds such as alkaloids, terpenoids, reducing sugars and anthroquinones, detected by chemical analyses [74]. In similar works, two strains of *Vibrio* (P1Ma8 and P1Ma5) and several *Bacillus* sp. isolated from the sponges *Phorbas tenacior* and *Tedania anhelans*, respectively, displayed enhanced free radical scavenging activity evaluated by DPPH assay [75,76]. The anti-oxidant properties of a bioactive compound (Pyrrolo[1,2-a]pyrazine-1,4-dione, hexahydro-C_7_H_10_N_2_O_2_) extracted from a sponge-derived *Bacillus* sp. (Lakshadweep archipelago in India) was also studied using DPPH assay, nitric oxide (NO) and hydrogen peroxide (H_2_O_2_) scavenging activity, and total reducing power. The active compound was capable of scavenging H_2_O_2_ in a dose-dependent manner. Moreover, IC_50_ for NO and DPPH inhibition was 41.70 μg/mL and 15.025 μg/mL, respectively [77].

Bioactivity screening of one hundred bacterial bionts isolated from several Indian sponges led to the isolation of the GUVFCFM-3 strain, identified as *Chromohalobacter israelensis*. In particular, the methanol extract showed a significant percentage of DPPH (67.83%) and superoxide (65.87%) scavenging activities [78]. DPPH tests and quantification of total phenolic content (TPC) were also used to evaluate the anti-oxidant activity of *Pseudomonas* sp. extract associated to the marine sponge *Hyrtios aff. erectus* from the Red Sea. In particular, DPPH assay showed a 100% of inhibition at all quantities tested (50, 25, 12.5 and 6.25 mg) [79]. Moreover, Vijayan et al. [80] demonstrated that bacteria associated to darkly pigmented sponges (*Haliclona pigmentifera*, *Sigmadocia pumila*, *Fasciospongia cavernosa*, *Spongia officinalis* and *C. diffusa*) collected from the Gulf of Mannar in Indian ocean produced non-cytotoxic melanin, with anti-oxidant and photoprotective activities. Among bacterial strains demonstrating high production of melanin, *Vibrio alginolyticus*, isolated from *Haliclona pigmentifera*, *Sigmadocia pumila* and *S. officinalis*, protected mouse fibroblast cells (L929) from UV-induced intracellular reactive oxygen stress (IC_50_ = 9.0 μg/mL) and exerted no cytotoxicity on L929 cells and brine shrimps [80]. Sponge derived strains retrieved from Indonesian waters, HAL-08, HAL-13 and HAL-74 (*Haliclona* sp.) as well as PTR-21 (*Petrosia* sp.), were evaluated using the DPPH and ABTS (2,2′-azinobis3-ethylbenzothiazoline-6-sulfonate) methods. Among the isolates tested, the highest anti-oxidant activity was revealed by the crude extract of HAL-08 with IC_50_ values of 17.10 and 59.39 μg/mL for DPPH and ABTS radicals, respectively. In addition, PTR-21 appeared to be the most potent anti-aging agent tested on the viability of *Schizosaccharomyces pombe* [81]. The anti-oxidant activity of bacteria PTR-08, PTR-40, PTR-41, and PTR-47, identified as *Pseudomonas* sp., was also evaluated. PTR-08 extract exhibited the highest anti-oxidant properties with IC_50_ values of 9.25 and 235.53 μg/mL for DPPH and ABTS radicals, respectively. Interestingly, PTR-08 modulated yeast longevity of *Schizosaccharomyces pombe* promoting the anti-oxidant defence mechanisms correlated with intracellular oxidative stress [82]. The same authors examined the extract of another Indonesian bacteria (*Pseudoalteromonas flavipulchra*, named STILL-33) associated to the sponge *Stylotella* sp. STILL-33, which exhibited a high DPPH and ABTS degrading activity with IC_50_ values of 7.80 μg/mL (DPPH) and 31.50 μg/mL (ABTS) [83].

Some works, together with the anti-oxidant capabilities, evaluated the growth inhibition activity of specific pathogens commonly involved in skin infections. For instance, a chlorinated quinolone, Ageloline A, isolated from *Streptomyces* sp. SBT345, a bacterial symbiont of the Mediterranean sponge *Agelas oroides*, was investigated for its radical scavenging and anti-microbial properties. This compound exhibited anti-oxidant potential on a human leukemic cell line (HL-60) and was further able to reduce oxidative stress and genomic damage induced by 4-nitroquinoline-1-oxide (NQO). Moreover, Ageloline A inhibited the growth of *Chlamydia trachomatis* in a dose-dependent manner with an IC_50_ value of 2.14 μg/mL [84]. Anti-microbial activities against *E. coli* MTCC-1687, *P. aeruginosa* MTCC-1688, *B. subtilis* MTCC-441 and *S. aureus* MTCC-737 were also observed from a GSA10 strain associated to the sponge *Halichondria glabrata* (West coast of Mumbai, India). In addition, anti-oxidant properties were detected using DPPH scavenging and Total Radical-trapping Anti-oxidant Parameter (TRAP) assay. In particular, through TRAP assay, the GSA10 acted as peroxyl scavengers, and the percentage of inhibition was proportional to the GSA10 concentrations [85]. In a recent work, the crude methanolic extract and the fractions of *Bacillus* 2011SOCCUF3 strain isolated from the sponge *S. officinalis* (Cortiou and Riou, France) exhibited anti-oxidant and anti-microbial activities. In particular, DPPH assay showed a dose-dependent scavenging activity, with a percentage inhibition of 38.9-49.1% (10–50 mg/mL), and agar-well diffusion method revealed a high inhibitory effect against *C. albicans* at a concentration range of 2.5–20 mg/mL [86].

Anti-aging and skin whitening properties from the crude extracts of bacterial symbionts from *Scopalina hapalia* (South-east coasts of Mayotte) were evaluated on several targets, including elastase, tyrosinase, catalase, sirtuin 1 (Sirt1), cyclin-dependent kinase 7 (CDK7), fyn kinase, and proteasome [66]. In particular, the isolate SH-82 (*Micromonospora fluostatini*) exhibited sufficient inhibition of elastase activity, whereas SH-89 exerted significant anti-melanogenic properties by tyrosinase inhibition (58.33%). The most potent activators of Sirt1 activity were shown by SH-82 and SH-100 (*Bacillus licheniformis*) extracts. Moreover, four *Bacillus* strains and three extracts of *Salinispora arenicola* exhibited appreciable anti-oxidant and CDK7 inhibitory activities, respectively. Surprising results were reported from *S. arenicola* (SH-78-EA-SM) and *B. licheniformis* (SH-04-EA-SM), inhibiting Fyn activity at the three concentrations tested (0.033, 0.0033 and 0.00033 µg/mL). In contrast, the crude extracts of SH-45, SH-54, SH-78, and SH-99 exhibited a slight activity, detected only at the highest concentration (0.033 µg/mL). On the whole, the authors have proposed these sponge-derived bacteria as suitable sources of new skin whitening and anti-aging agents [66].

As mentioned above (see the introduction section), microbial biosurfactants displayed suitable properties for skincare formulations [57]. For instance, Dhasayan et al. [87] evaluated the moisturizing features of several strains isolated from the Indian sponge *C. diffusa*. In particular, the MB-30 (*Halomonas* sp.) and MB-I9 (*Alcaligenes* sp.) exhibited the highest emulsification activity after 48 h of incubation, whereas MB-7 (*Bacillus subtilis*) and MB-101 (*Bacillus amyloliquefaciens*) isolates showed the same properties after 72 h of incubation, suggesting that the bioactive compounds are probably secreted during the stationary phase of growth [87]. Moreover, in a recent work, a bacterial strain (*Bacillus niabensis*, My-30) associated to the sponge *Mycale ramulosa* (Gulf of California) showed a clear activity in the collapsing drop test and emulsification properties with high stability for 24 h, compared to the control (Sodium Dodecyl Sulfate, SDS). Moreover, supernatants of My-30 demonstrated a promising antifouling activity, with Minimum Inhibitory Concentration (MIC) values of 1–2% (*v/v*), against *Bacillus subtilis*, *Micrococcus* sp., and *Sagittula stellata* [88].

The cosmeceutical compounds discussed above are listed in Table 1.

### 2.2. Fungi

So far, several fungal isolates showed pharmacological activities, including anti-bacterial, anti-oxidant, anti-proliferative, and so on [89,90,91,92,93]. Since 1998, a research group published some results about an amylase isolated from the fungus *Mucor* sp. associated to the sponge *Spirastrella* sp. Amylases are enzymes involved in cellular and metabolic pathways, useful for the pharmaceutical, cosmeceutical (as a detergent), and nutraceutical industries [94]. A well-known example is Circumdatin, a benzodiazepine alkaloid that has been isolated from a fungus of the genus *Exophiala* (Bogil Island, Korea). Due to its UVA photo protection, with higher effectiveness than common sunscreen agents, this molecule is now used in commercial products [95]. Similarly, in a recent work, some fungal meroterpenoids described from the fungus *Penicillium brasilianum* WZXY-m122-9, were isolated from an unidentified marine sponge collected in the South of China. Among the chemically characterized compounds, Brasilianoid A was capable of up-regulating *filaggrin* and *Caspase-14* expression and increasing the viability (up to 77%) of UVB irradiated human keratinocytes (HaCaT). The authors considered this compound to be a promising candidate for the treatment of dermatological diseases and skin protection from UVB damages [96].

Concerning the anti-oxidant activity, two extracellular polysaccharides, ENP1 and ENP2, were isolated from the fermentation fluid of the sponge-derived marine fungus *Epicoccum nigrum* (Hainan, China). Both molecules exhibited, in vitro, a slight anti-oxidant capacity by hydroxyl, superoxide, and DPPH assay, with low half maximal effective concentration (EC_50_) values (280–1570 µg/mL). However, ENP2 was found to be the most active, since a higher radical scavenging activity was measured at all concentrations tested [97]. The same properties were detected from an aromatic polyketide isolated from *Aspergillus versicolor*, a fungus cultured in laboratory conditions after rinsing some tissues excised from the Korean sponge *Petrosia* sp. (Jeju Island in South Korea). By comparing it to standard anti-oxidants, this compound displayed anti-oxidant properties at increasing concentrations (5–100 µg/mL) by DPPH assay and inhibition of lipid peroxidation, higher than butylated hydroxytoluene (BHT) [98]. Bioassay-guided fractionations led to the isolation of other anti-oxidant compounds from the sponge-derived fungus *Penicillium citrinum* SpI080624G1f01 (Ishigaki Island, Japan). The DPPH radical scavenging activity of a sorbicillinoid derivative, named JBIR-124, was found particularly interesting, with an IC_50_ value of 13 µg/mL [99]. In a similar work, DPPH combined to Thiobarbituric acid (TBARS) and NO assay, showed significant anti-oxidative and anti-inflammatory activities of the crude extracts obtained from three fungi, *Chaetomium globosum*, *Gymnascella dankaliensis* and *Nigrospora oryzae*. These fungal strains were isolated from the sponge *Hippospongia communis*, collected between the West coast of Alexandria and the borders of Libya. In particular, *C. globosum* and *G. dankaliensis* displayed a significant inhibition of lipid peroxidation (93%) and DPPH scavenging activity (59%), respectively, whereas *N. oryzae* was the most effective in inhibiting NO species [100]. Extensive chemical analyses also allowed the identification of more than twenty Spiro-Phthalides and Isocoumarins from the fungus *Setosphaeria* sp. SCSIO41009 associated to the sponge *Callyspongia* sp. retrieved from Chinese waters. Among them, 7-O-demethylmonocerin evidenced a strong scavenging activity on DPPH radicals, with IC_50_ value (11.2 µg/mL) comparable to ascorbic acid [101]. From the ethyl acetate extract of the Chinese strain *Aspergillus europaeus* WZXY-SX-4-1, isolated from the marine sponge *Xestospongia testudinaria*, six polyketide derivatives were separated through chromatographic method. Bioactivity screening showed that three Benzophenones exhibited the most potent scavenging activity against DPPH radicals (IC_50_ = 1.7–5.4 µg/mL) as compared to the positive control (trolox) [102]. A fungus species identified as *Aspergillus unguis* RSPG_204 was isolated from the sponge *Agelas* sp. (Hurghada coast, Red Sea, Egypt). The mycelia extract and culture supernatants exhibited significant superoxide anion scavenging activity, while extremely low anti-tyrosinase capabilities were detected. Interestingly, the biological activity was corroborated by chemical analyses revealing several bioactive compounds in the supernatants of static cultures and mycelial extract [103]. Recently, a marine fungus of the same genus (*Aspergillus terreus*), living as symbiont of the marine sponge *Phakellia fusca*, was found to produce four butenolide derivatives. DPPH assay revealed moderate anti-oxidant properties (IC_50_ = ~14–36 µg/mL) with promising application in the cosmeceutical field [104].

Henriquez et al. [105] sampled eleven marine sponges from the Fildes Bay (King George Island, Antarctica), belonging to the genera *Tedania* sp., *Hymeniacidon* sp., *Dendrilla* sp., and three unidentified ones grouped in the order Poecilosclerida. Through sequence analysis of the ITS1-5.8S-ITS2 region, 24 genotypes linked to the four taxonomic classes Leotiomycetes, Dothiodeomycetes, Eurotiomycetes, and Sordiaromycetes were identified. Among the fungal extracts tested for their anti-bacterial activity on *P. aeruginosa* and *S. aureus* ATCC25922, more than half of them showed inhibitory activity against one of the bacterial strains analyzed. Interestingly, several fungal isolates with the same ITS genotype showed completely different activities. The anti-oxidant capacity, evaluated for all the extracts, revealed a wide range of activities that ranged from very low to extremely high for three isolates of the genus *Epicoccum* (F09T15-3, F09-T15-6) and an unknown one (F09-T18-16) [105].

Moderate anti-bacterial and anti-oxidant activities were instead observed from furan, cyclopentenone and tyrosol derivatives isolated from the fungus species *Hypocrea koningii* PF04 (South China) and Acremostrictin, a tricyclic lactone identified from the culture broth of *Acremonium strictum* (Gagu-do, Korea) [106,107,108]. In particular, Hypofurans A/B, Hypocrenones A/B/C, and Hypocrol A displayed low inhibitory activity on *S. aureus* ATCC25923 and *E. coli* [107,108]. Moreover, Hypocrol A and Trichodenol B revealed a slight anti-oxidant capacity, with IC_50_ values of 48.5 and 97.4 μg/mL, respectively [108]. Similarly, Acremostrictin reduced DPPH radicals on H_2_O_2_-induced HaCaT cells in a dose-dependent manner with IC_50_ of 529.2 μg/mL [106]. Since low activities have been detected, these compounds clearly reported unsuitable cosmeceutical features.

The bioactivity of twenty-two fungi associated to several sponge species (*Agelas citrina*, *Stelligera rigida*, *Oscarella lobularis*, *Celtodoryx girardae*, *Madracis miriabilis*, *Cliona celata* and *Spongosorites difficilis*) collected from the Red Sea was also tested for their anti-microbial and anti-oxidant capacities. An anti-microbial assay was carried out upon agar plates containing the bacterial pathogens *S. aureus*, *P. aeroginosa* and *C. albicans*. Among fungi under analysis, the most promising anti-microbial activities against all pathogens tested were ascribed to *Aspergillus oryzae* and *Cladosporium cladosporioides*. Regarding the anti-oxidant properties, the fresh mycelium was found more effective than the culture filtrate extract, with *Aspergillus fumigatus* reporting the highest percentage of DPPH scavenging activity (59.7%) [109]. A different study conducted on the sponge *Amphimedon* sp. (Yongxin Island, China) brought to the isolation of the fungus *Peyronellaea glomerata*. Chromatographic separation of the ethyl acetate extract revealed five Isocoumarins, Peyroisocumarins A-D and Isocitreoisocoumarinol, plus thirteen analogs. The anti-bacterial assays were applied to different organisms, including *S. aureus* and *E. coli*. In particular, Alternariol slightly inhibited the growth of *S. aureus* (MIC = 16 µM). On the other hand, through Antioxidant Response Element (ARE)-driven luciferase reporters, a significant regulation of the nuclear factor E2-related factor 2 (Nrf2), a transcription factor that responds to oxidative stress, was observed in Peyroisocumarins A and B with chlorination at side chain. Hence, these two compounds were suggested by the authors as potential leads for anti-oxidant agents [110].

Several biological activities were also found in the extracts obtained from the sponge-derived *Aspergillus sydowii* strain W4-2 and an unidentified fungus named FS1 (Red Sea, Egypt). The supernatant of crude extracts obtained from fungal static cultures showed a high DPPH free radical scavenging activity, plus a moderate tyrosinase inhibitory capacity of *A. sydowii*. Moreover, FS1 demonstrated anti-bacterial properties against *S. aureus*, *C. albicans* and *P. aeruginosa* [111]. Interestingly, another *A. sidowii* strain isolated from the Indonesian sponge KN-15-3 also demonstrated significant anti-bacterial activity on Multi-Drug Resistant *S. aureus* and *E. coli* bacteria [112]. Contrary to the results reported by El-Hady and collaborators [103,111], the ethyl acetate extract of fungal strains (*Penicillium* sp., *Aspergillus niger* and *Trichophyton megninii*) isolated from another Indonesian sponge (*Haliclona fascigera*) displayed considerable anti-tyrosinase activity. In particular, all fungi inhibited tyrosinase functionality, and only one strain of the genus *Penicillium* was found extremely active (IC_50_ = 26 µg/mL). Overall, these species were pointed out as potential sources of tyrosinase inhibitors and skin-whitening agents [113].

The sole anti-biofilm activity against *S. epidermidis* was instead evaluated in the dipeptide cis-cyclo(Leucyl-Tyrosyl) isolated from the symbiotic ascomycete *Penicillium* sp. F37. Interestingly, the dipeptide was able to reduce biofilm formation (~60–85%) at 0.25, 0.5 and 1 mg/mL without blocking bacterial growth, which was then inhibited at higher doses (2 mg/mL). The anti-biofilm activity was corroborated by Scanning Electron Microscopy (SEM), showing a clear attachment of bacteria in untreated biofilms with a visible production of exopolysaccharides (EPS) [114].

The anti-oxidant and anti-aging properties of the culture filtrate (ACCB) obtained from the sponge-associated *Aspergillus chavalieri* TM2-S6 (Tel Aviv-Jaffa, Israeli Mediterranean coast) were also tested on primary normal human dermal fibroblasts (NHDF) through bioassays and molecular approaches [115]. Chemical analyses on the ACCB ethyl acetate extract revealed two abundant compounds, named Tetrahydroauroglaucin and Flavoglaucin. To correlate the chemical composition to the biological activity of ACCB, ATP assay was performed on H_2_O_2_ treated NHDF cells. Experimental results showed that the incubation with ACCB at 0.05 µg/mL increased cell viability, in comparison to the samples without ACCB. Gene expression analysis corroborated the anti-oxidant capacity of ACCB through the up-regulation of *glutathione peroxidase-1* (*GPX-1*), *superoxide dismutase-1* (*SOD-1*) and *erythroid 2 like 2* (*NRF2*) genes. Moreover, ACCB promoted cell proliferation and extracellular matrix organization, since the expression levels of six key genes involved in these processes, *collagen type I alpha 1 chain* (*COL1A1*), *collagen type III alpha 1 chain* (*COL3A1*), *matrix metallopeptidase 14* (*MMP14*), *CD44 molecule* (*CD44*), *vascular endothelial growth factor A* (*VEGFa*) and *transforming growth factor beta 3* (*TGFB3*), significantly increased. Interestingly, the mRNA levels of *sirtuin 1* (*SIRT1*) and *sirtuin 2* (*SIRT2*), implicated in skin aging, were also found up-regulated in H_2_O_2_-induced NHDF cells. Combining these results, the authors concluded that ACCB stimulated cell proliferation, anti-oxidant response and extracellular matrix organization as well as reduced aging, thus proposing ACCB as a perfect candidate for the formulation of cosmetic products [115].

The aforementioned biological activities of fungi isolated from marine sponges are schematically reported in Table 2.

## 3. Sponges

The isolation of sponge-associated microbiota was reported as a complicated step within drug discovery pipelines, since the majority of them are uncultivable in laboratory conditions [116]. For this reason, a considerable amount of literature focused on activity screenings without investigating whether the bioactive metabolites were produced by symbiotic bacteria or the sponge hosts.

Among the natural compounds with possible applications in cosmeceutical fields, free radical scavenging activity was particularly retrieved. Avarol and its derivatives, isolated from the Mediterranean sponge *D. avara* (Bay of Naples, Italy), were investigated for their anti-oxidant, anti-inflammatory and anti-proliferative properties, and the results were compared to the well-known activity of Avarol. In particular, DPPH assay and ROS generation in stimulated human neutrophils revealed that avarol-3′-thiosalicylate (TA) was the most active (DPPH, IC_50_ = 15.9 µg/mL), with ROS scavenging capability even higher (IC_50_ = 1.2 µg/mL) when compared to Avarol (IC_50_ = 1.7 µg/mL). Interestingly, the same compound also inhibited prostaglandin E2 (PGE_2_) production in HaCaT cell line. Therefore, the authors suggested that the sponge derived compound could potentially block the inflammatory events associated to psoriasis [117]. This latter hypothesis was later explored through in vitro experiments revealing a considerable reduction of (i) PGE_2_ and (ii) TNF-α levels in human monocytes and (iii) NFκB binding to DNA in HaCaT cells. Since a crosstalk between TNF-α and NFκB was detected in patients with psoriasis, a role of TA compound in the treatment of psoriatic patients has been corroborated [118].

The ethylacetate extracts from the sponges *Rhabdastrella globostellata* and *Spirastrella inconstans* (Gulf of Mannar) were investigated for their anti-oxidant activity in vivo at different concentrations (2, 4, 6, 8 and 10 mg/kg). The oral administration in rats of the sponge extracts increased the hepatic activity of superoxide dismutase (SOD), catalase (CAT) and glutathione peroxidase (GPx) enzymes [119]. Moreover, the dichloromethane and methanol extracts of the sponges *Fascaplysinopsis reticulata*, *Callyspongia siphonella*, *Niphates furcata*, *Callyspongia* sp., *Callyspongia clavata* and *Pseudosaberites clavatus* harvested from the North coast of the Persian Gulf were also investigated for their free radical scavenging capabilities by DPPH and Hydroxyl Radical Scavenging assays. In particular, the methanol extract of the sponge *P. clavatus* displayed the best anti-oxidant activity on DPPH (IC_50_ = 234 μg/mL), while both extracts of *N. furcata* and *F. reticulate* were clearly inhibited OH radicals (~70–80%) [120]. Similarly, the anti-oxidant activity was evaluated in the total extracts of six sponges collected from Indonesia using DPPH assay. The authors found that *Aaptos suberitoides* induced the highest activity (IC_50_ < 3 × 10^4^ μg/mL), while *F. reticulata*, *Acanthella* sp., *Petrosia contignata* and *Xestospongia exigua* exerted only a slight anti-oxidant effect with IC_50_ values less than 1 × 10^5^ µg/mL [121]. The methanol extracts of eleven sponge species collected from six geographical sites in Turkey were evaluated for their anti-oxidant properties through DPPH, NO and superoxide radical scavenging activities. The DPPH and superoxide assays revealed a significant dose-dependent radical scavenging activity, with the sponge *Dysidea avara* being the most promising among all specimens under analysis (DPPH, IC_50_ = 92.8 µg/mL; O_2_^−^, 34.1 µg/mL). Concerning NO radicals, a moderate activity was recorded, with the sole methanolic extract of *Ciocalypta carbolloi* displaying anti-oxidant capacities (700.7 µg/mL) that were higher when compared to the control (quercetin). Interestingly, the authors noticed that the biological activity of sponge extracts was clearly correlated to the location, since the samples collected from Kemer revealed the highest anti-oxidative properties [122]. On the contrary, Botic et al. [123] observed an anti-oxidative capability of different Antarctic sponges of the genus *Latrunculia* through photochemiluminescence assay. The significant variation among samples was explained by a probable changing in the symbiotic community, which was not influenced by the geographical site but rather was species specific [123]. Puupehenol, a meroterpenoid isolated from the organic extract of the Hawaiian Deep-Water sponge *Dactylospongia* sp. was found to be both an anti-oxidant and anti-microbial compound. In fact, a significant radical scavenging activity, detected by Ferric Reducing Antioxidant Power (FRAP) Assay, and a moderate growth inhibition of the Gram-positive bacteria *Staphylococcus aureus* was detected [124]. Anti-oxidant activity was also demonstrated in the crude extract of the sponge *Ircinia spinulosa* collected from the Atlantic Moroccan coast. DPPH assay revealed considerable free radical scavenging capabilities (25.25%) of the crude extract, together with a content of polyphenols, flavonoids and tannins [125]. Tannins and flavonoids were also found in the sponges *A. suberitoides*, *Dactylospongia elegans*, *Stylissa massa* and *Haliclona* sp. (Red Sea, Egypt). The anti-oxidant activity, evaluated by the phosphomolybdenum method, revealed that, among the samples analyzed, the hexane extract of *D. elegans* and the ethyl acetate extract of *A. suberitoides* induced the highest anti-oxidant properties in comparison to the ascorbic acid [126]. Recently, DPPH and 2.2′-azino-bis(3-ethylbenzothiazoline-6-sulfonic acid assays also displayed a significant anti-oxidant activity (93% and 99%, respectively) of *H.* aff. *erectus* (Red Sea, Egypt) extract at 1 mg, plus a considerable content of carotenoids (1.976 mg/g) [127]. Moreover, 141 extracts of other sponge samples collected from Mauritius were investigated by DPPH and FRAP assays. The two sponges *Axinella donnani* and *Pseudosuberites* sp. were found the most promising, with significant radical scavenging activities, measured as 92.15% (DPPH) and 10.57 Fe^2+^/g of extract (FRAP), respectively [128]. The anti-oxidant capacity was also analyzed in the protein extract and the ammonium sulfate fractions of the sponge *Niphates* sp. collected from the sponge reefs of Spermonde waters (South Sulawesi). Among the samples investigated, the DPPH radical scavenging was mostly observed in the crude extract (IC_50_ = 5.05 µg/mL), probably due to a higher glutathione content enhancing the anti-oxidant potentialities [129].

The anti-inflammatory and photoprotective role of Topsentin, a bis(indolyl)imidazole alkaloid identified from the Korean sponge *Spongosorites genitrix*, was evaluated by a multi-approach study [130]. Human keratinocytes HaCaT cells were irradiated with UVB rays and then treated with increasing concentrations of Topsentin (1.25, 2.5, 5 and 10 µM) to measure the protective effect of the sponge derived compound. Western blot and ELISA assay revealed a dose-dependent reduction of cicloxygenase 2 (COX-2) proteins and PGE_2_ in cell supernatants (IC_50_ = 0.4 µg/mL) after Topsentin treatment. These data were corroborated by gene expression analyses, showing a significant down-regulation of *COX-2 miR-4485* (a miRNA involved in UVB-induced skin inflammation) and the correlated *tumour necrosis factor alpha induced protein* (*TNF-α IP2*). As expected, Topsentin exposure also reduced PGE_2_ levels in human skin models and visibly restored the tissue layer after the damage of UVB radiation [130].

Topic formulations with skin whitening properties have also found huge applications in the cosmetic industry. Concerning sponge-derived compounds, experiments of immunofluorescence on murine melanoma B16 cells treated with the anti-tumour compound Geoditin A, isolated from the sponge *Geodia japonica* (South China Sea), revealed anti-melanogenic and skin whitening properties. Increasing concentrations (0.6, 1.25 and 5 μg/mL) of Geoditin A induced a dose-dependent reduction of melanin within the cytosol and Golgi apparatus, and, similarly, a depletion of tyrosinase was observed into the endoplasmic reticulum (ER). The decrease of tyrosinase activity after Geoditin A treatment was corroborated through the detection on immunoblotting of melanogenic proteins [131]. Similarly, Gagunin D (GD) (see Figure 1), a diterpenoid isolated from the sponge *Phorbas* sp. (Gagu-Do, Korea), was revealed as a potent anti-melanogenic compound [132]. In particular, treatments of GD at increasing concentrations significantly reduced the production of melanin (IC_50_ = 5.7 µg/mL) in Melan-a cells, with higher effects in comparison to the commercial skin whitening agent arbutin. This result was also confirmed by Real time qPCR on melanogenesis-related genes revealing a significant down-regulation of *PAX3*, *SOX10*, *MITF*, *tyrosinase*, *TRP-1* and *TRP-2*. Moreover, GD exposure at 10 µM on UVB irradiated human skin models demonstrated a considerable reduction of melanin biosynthesis [132].

The methanol, ethanol and hexane extracts obtained from *Acanthella cavernosa*, a sponge collected from Bali (Indonesia), were rather explored for anti-microbial and anti-biofilm properties against *Propionibacterium acnes* [133], a common pathogen inducing the inflammatory events connected to acne issues. In particular, the ethanol extract displayed MIC and Minimum Bactericidal Concentration (MBC) values of 125 and 250 μg/mL, respectively, and a considerable inhibition of *P. acnes* biofilm at 250 μg/mL (45%). These results, combined to a slight anti-oxidant activity, suggested a possible application of these sponge extracts as cosmetic ingredients for preventing acne infections [133]. The anti-microbial activity of seven sponge extracts was also evaluated through agar well diffusion on *S. epidermis*, *S. aureus* and *P. aeruginosa* [134], three microbes that normally constitute skin microflora. Five sponge samples retrieved from Indian waters revealed anti-microbial activity, with *Neopetrosia exigua* extract being the most promising against target organisms plus *C. albicans* showing significant anti-fungal properties. Moreover, all methanol extracts exerted anti-oxidant effects by DPPH assay, particularly *Hyrtios erecta* (IC_50_ = 32.5 μg/mL), followed by *N. exigua* (IC_50_ = 36.6 μg/mL) and *X. testudinaria* (IC_50_ = 46.7 μg/mL) species [134]. In a recent work, bioassay-guided fractionations from the CH_2_Cl_2_-MeOH extract of the sponge *Haliclona* sp. collected in the Indian Ocean led to the identification of several long-chain highly oxygenated polyacetylenes, named Osirisynes A, B, E, G, H and I. These latter compounds were all tested for catalase and sirtuin 1 activation and CDK7, proteasome, Fyn kinase, tyrosinase, and elastase inhibition, which are considered suitable targets for studying aging-related diseases. In particular, Osirisyne B was found the most effective, with a significant blockage of Fyn kinase (IC_50_ = 14.7 µg/mL), CDK7 kinase (IC_50_ = 7.3 µg/mL), and proteasome (IC_50_ = 0.2 µg/mL) [135].

Marine natural compounds with wound healing properties were also identified as suitable sources for cosmetic manufacturing. Fibroblasts normally produce several compounds in the extracellular matrix, such as glycosaminoglycans (GAGs) or collagen, with the specific capability to adsorb the excessive exudate within tissue wounds and promote skin repair [136]. Advances in chemical extraction methods allowed the isolation of collagen and other similar substances from marine invertebrates, including sponges [137]. One of the first studies observed that collagen, extracted from the sponge *Condrosia reniformis* (Aegean Sea), slightly influenced skin pH and hydration, revealing promising results [138]. Then, in a different research, four sponge samples, *Spongia lamella*, *Spongia officinalis*, *Hippospongia communis* and *Sarcotragus spinosulus* collected from Sardinian beaches (Western Mediterranean Sea), were also found to produce considerable quantities of natural GAGs with good water adsorbing capabilities [139]. Moreover, a recent work evaluated the anti-oxidant, photoprotective and wound healing properties of collagen hydrolysate (MHC) fractions from the Mediterranean sponge *C. reniformis* [140]. DPPH and Nitro Blue Tetrazolium (NBT)/riboflavin assays displayed a high ROS and superoxide anion scavenging activity at 50 µg/mL and 100 µg/mL compared to controls. In addition, the exposure to MHC fractions increased the mRNA levels of *collagen 1A* (*Col1A*) in L929 murine fibroblasts and enhanced cell growth in UV flashed L929 (~8–40%) and HaCaT (~14–32%) cells, depending on the UV dose. “Scratch” tests showed good skin repair properties, particularly on keratinocytes, where cell proliferation near the wound edges was clearly visible at 6 h and 24 h of treatment, with ~22% of wound extension [140].

The chemical compounds and biological activities described in this section are summarized in Table 3.

## 4. Conclusions

Despite the fact that Porifera has historically been considered as a basal phylum among marine organisms, researchers have discovered that they can represent a real possibility for improving the life quality of entire human communities in the future. In the present review, we analyzed several bioactive compounds isolated from sponges and their associated microorganisms and symbionts with suitable features for cosmeceutical applications. We emphasized that those compounds isolated from sponges might derive from their symbiotic community. Nevertheless, it must be considered that bacteria and fungi isolated and cultivated in a laboratory are merely marine opportunistic microbes and, with high probability, not specifically associated to sponges.

The above-mentioned compounds exerted several activities such as anti-oxidant, anti-inflammatory, anti-microbial, anti-aging, skin-whitening, wound healing and moisturizing properties. In particular, most of them exhibited anti-oxidant properties, whose biological function might be extremely useful for preventing skin aging. However, this could be due to the experimental procedures adopted, since free radical scavenging properties are normally detected through colorimetric assays (e.g., DPPH), which are relatively fast, cheap, and easy to apply.

Among thirty-seven papers published in the last ten years on bacteria and sponges, a considerable number of compounds were purified and chemically characterized, such as Ganunin D, Osirisynes, Topsentin and Ageloline A, although the majority of the works relied on the investigation of the crude extracts. On the contrary, almost all papers on fungi reported isolated compounds, as in the case of 7-O-demethylmonocerin, Brasilianoid A, Tetrahydroauroglaucin and Flavoglaucin, which were then correlated to the biological activity. Among bacterial and fungal symbionts, *Bacillus*, *Penicillium* and *Aspergillus* were found to be the most abundant genera with interesting features, since the crude extracts and/or molecules displayed suitable biological activities. Moreover, an important finding may be represented by the isolation of non-toxic and bio-compatible melanin from sponge associated bacteria for its promising application in UV-protective products. Regarding sponges, the relevance of *C. reniformis*, which is due to the discovery of a natural collagen and which has potential wound healing properties, must also be remarked upon.

Despite there being numerous promising candidates of bioactive and beneficial compounds investigated in sponges and their symbionts, only a few examples of commercial products were found, as in the case of Circumdatins, a fungus-derived molecule which is now used in sunscreen formulations. Several steps are still extremely long and not well standardized, so more efforts are needed to improve the (i) isolation and characterization of the products (sometimes very difficult due to the uniqueness of the chemical structures of some compounds of marine origin), (ii) chemical modification techniques, (iii) evaluation of their pharmacological properties and safety aspect, and (iv) estimation of the product quality. For this reason, the pipeline that goes from the isolation of a potential cosmetic product to the evaluation of its safety for human usage should certainly be improved, at least as concerns those molecules isolated from sponges and their symbionts.

## Figures and Tables

**Table 1:** Bacterial source, sponge host, compound/extract, biological activity, and reference are reported.

Source	Sponge Host	Extract/Compound	Biological Activity	Reference
*R. squalenifaciens*	*Halichondria okadai*	Diapolycopenedioic acid xylosyl ester A	Anti-oxidant	[72]
*Streptomyces*	*C. diffusa*	Carotenoid extracts	Anti-aging	[71]
*Micromonospora* sp. RV115	*A. aerophoba*	Diazepinomicin	Anti-oxidant	[73]
*Virgibacillus* sp.	*C. diffusa*	Ethyl acetate extracts	Anti-oxidant	[74]
*Vibrio* (P1Ma8 and P1Ma5)	*P. tenacior*	CH_2_Cl_2_/MeOH (1:1) extracts	Anti-oxidant	[75]
*Bacillus* sp.	*T. anhelans*	Ethyl acetate extracts	Anti-oxidant	[76]
*Bacillus* sp.	Sponges from Lakshadweep archipelago	Pyrrolo[1,2-a]pyrazine-1,4-dione, hexahydro-C_7_H_10_N_2_O_2_	Anti-oxidant	[77]
*Halomonas* sp. MB-30 and *Alcaligenes* sp. MB-I9	*C. diffusa*	Isolates of bacteria	Biosurfactants	[87]
*C. israelensis*	*Callyspongia fibrosa*	Ethyl acetate extracts	Anti-oxidant	[78]
*Streptomyces* sp. SBT345	*A. oroides*	Ageloline A	Anti-oxidant	[84]
*Pseudomonas* sp.	*H. aff. Erectus*	Ethyl acetate extracts	Anti-oxidant	[79]
*V. alginolyticus*	*H. pigmentifera*, *S. pumila* and *S. officinalis*	Melanin extracts	Anti-oxidant	[80]
GSA10	*H. glabrata*	Ethyl acetate extracts	Anti-oxidant	[85]
HAL-08, HAL-13, HAL-74 and PTR-21	*Haliclona* sp. and *Petrosia* sp.	Ethyl acetate extracts	Anti-oxidant	[81]
PTR-08, PTR-40, PTR-41, and PTR-47	*Petrosia* sp.	Ethyl acetate extracts	Anti-oxidant	[82]
*P. flavipulchra* STILL-33	*Stylotella* sp.	Ethyl acetate extracts	Anti-oxidant and anti-aging	[83]
*B. niabensis* (My-30)	*M. ramulosa*	Isolates of bacteria	Biosurfactants	[88]
*Bacillus* 2011SOCCUF3	*S. officinalis*	Methanol crude extracts	Anti-oxidant and anti-microbial	[86]
SH-82 (*M. fluostatini)*	*S. hapalia*	Ethyl acetate and methanol extracts	Anti-oxidant	[66]

**Table 2:** Fungal source, sponge host, compound/extract, biological activity and reference are reported.

Source	Sponge Host	Extract/Compound	Biological Activity	Reference
*Exophiala*	*H. panicea*	Circumdatin	Anti-UV	[95]
*A. strictum*	Unidentified marine sponge of the class Choristida	Acremostrictin	Anti-microbial and anti-oxidant	[106]
*E. nigrum* JJY-40	Unidentified marine sponge	ENP1, ENP2	Anti-oxidant	[97]
*A. versicolor*	*Petrosia* sp.	Aromatic polyketide	Anti-oxidant	[98]
*P. citrinum* SpI080624G1f01	Unidentified marine sponge	JBIR-124	Anti-oxidant	[99]
*C. globosum, G. * *dankaliensis* and *N. oryzae*	*H. communis*	Ethyl acetate extract	Anti-oxidant and anti-inflammatory	[100]
*Penicillium* sp. F37	*A. corrugata*	Cis-cyclo(Leucyl-Tyrosyl)	Anti-biofilm	[114]
*A. sydowii* strain W4-2 and unidentified fungus FS1	*Agelas* sp. and *Amphimedon viridis*	Crude extract of static cultures	Anti-oxidant, anti-tyrosinase and anti-microbial	[111]
F09T15-3, F09-T15-6, F09-T18-16	*Tedania* sp., *Hymeniacidon* sp., *Dendrilla* sp. and three Poecilosclerida	Ethyl acetate extract of culture medium	Anti-oxidant	[105]
*H. koningii* PF04	*P. fusca*	Hypofurans A/B and Hypocrenones A/B/C	Anti-microbial	[107]
*A. unguis* RSPG_204	*Agelas* sp.	Several metabolites from mycelia and culture supernatant extracts	Anti-oxidant and anti-tyrosinase	[103]
*H. koningii* PF04	*P. fusca*	Hypocrol A and Trichodenol B	Anti-microbial and anti-oxidant	[108]
*A. oryzae*, *C. cladosporioides*, *A. fumigatus*	*A. citrina*, *S. rigida*, *O. lobularis*, *C. girardae*, *M. miriabilis*, *C. celata* and *S. difficilis*	Mycelia and culture filtrate extracts	Anti-microbial and antioxidant	[109]
*P. glomerata*	*Amphimedon* sp.	Alternariol and Peyroisocumarins A and B	Anti-microbial and anti-oxidant	[110]
*A. sydowii*	KN-15-3	Culture extract	Anti-microbial	[112]
*A. europaeus* WZXY-SX-4-1	*X. testudinaria*	Eurobenzophenone C, 3-de-O-methylsulochrin and 14-de-Omethyl-5-methoxysulochrin	Anti-oxidant	[102]
*Setosphaeria* sp. SCSIO41009	*Callyspongia* sp.	7-O-demethylmonocerin	Anti-oxidant	[101]
*A. terreus*	*P. fusca*	Butyrolactone I, Butyrolactone II, 5-[(3,4-dihydro-2,2-dimethyl-2H-1-benzopyran-6-yl)-methyl]-3-hydroxy-4-(4-hydroxyphenyl)-2(5H)-furanone and Aspernolide A	Anti-oxidant	[104]
*P. brasilianum* WZXY-m122-9	Unidentified marine sponge	Brasilianoid A	Anti-UV	[96]
*Penicilium* sp., *A. niger* and *T. megninii*	*H. fascigera*	Ethyl acetate extract	Anti-tyrosinase	[113]
*A. chavalieri TM2-S6*	*Axinella* sp.	Tetrahydroauroglaucin and Flavoglaucin	Anti-aging and anti-oxidant	[115]

**Table 3:** Sponge species and genera, compound/extract, biological activity and reference are reported.

Sponge Species and Genera	Extract/Compound	Biological Activity	Reference
*C. reniformis*	Collagen	Wound healing	[138]
*D. avara*	Avarol-3′-thiosalicylate	Anti-oxidant and anti-inflammatory	[117]
*D. avara*	Avarol-3′-thiosalicylate	Anti-oxidant and anti-inflammatory	[118]
*R. globostellata* and *S. inconstans*	Ethyl acetate extracts	Anti-oxidant	[119]
*G. japonica*	Geoditin A	Skin whitening	[131]
*A. suberitoides*, *D. elegans*, *S. massa* and *Haliclona* sp.	Hexane and ethyl acetate extracts	Anti-oxidant	[126]
*F. reticulata*, *C. siphonella*, *N. furcata*, *Callyspongia* sp., *C. clavata* and *P. clavatus*	Dichloromethane and methanol extracts	Anti-oxidant	[120]
*F. reticulata*, *Acanthella* sp., *P. contignata, X. exigua* and *A. suberitoides*	Total extracts	Anti-oxidant	[121]
*D. avara* and *C. carbolloi*	Methanol extracts	Anti-oxidant	[122]
*Latrunculia bocagei* and *Latrunculia biformis*	Fatty acids extracts	Anti-oxidant	[123]
*Dactylospongia* sp.	Puupehenol	Anti-oxidant and anti-microbial	[124]
*A. cavernosa*	Methanol, ethanol and hexane extracts	Anti-acne	[133]
*Phorbas* sp.	Gagunin D	Skin whitening	[132]
*I. spinulosa*	Crude extract	Anti-oxidant	[125]
*S. lamella*, *S. officinalis*, *H. communis* and *S. spinosulus*	Glycosaminoglycans	Wound healing	[139]
*N. exigu, H. erecta* and *X. testudinaria*	Methanol extracts	Anti-oxidant and anti-fungal	[134]
*C. reniformis*	Collagen hydrolysate fractions	Wound healing and anti-oxidant	[140]
*Haliclona* sp.	Osirisynes A, B, E, G, H and I	Anti-aging	[135]
*S. genitrix*	Topsentin	Anti-inflammatory	[130]
*H.* aff. *erectus*	Crude extract	Anti-oxidant	[127]
*A. donnani* and *Pseudosuberites* sp.	Crude extract	Anti-oxidant	[128]
*Niphates* sp.	Protein extract and the ammonium sulfate fractions	Anti-oxidant	[129]

## Data Availability

Not applicable.
